# Arbovirosis and potential transmission blocking vaccines

**DOI:** 10.1186/s13071-016-1802-0

**Published:** 2016-09-23

**Authors:** Berlin Londono-Renteria, Andrea Troupin, Tonya M. Colpitts

**Affiliations:** Department of Pathology, Microbiology and Immunology, University of South Carolina, Columbia, South Carolina USA

**Keywords:** Transmission blocking vaccines, Arbovirus, Arthropod, Vectors

## Abstract

Infectious diseases caused by arboviruses (viruses transmitted by arthropods) are undergoing unprecedented epidemic activity and geographic expansion. With the recent introduction of West Nile virus (1999), chikungunya virus (2013) and Zika virus (2015) to the Americas, stopping or even preventing the expansion of viruses into susceptible populations is an increasing concern. With a few exceptions, available vaccines protecting against arboviral infections are nonexistent and current disease prevention relies on vector control interventions. However, due to the emergence of and rapidly spreading insecticide resistance, different disease control methods are needed. A feasible method of reducing emerging tropical diseases is the implementation of vaccines that prevent or decrease viral infection in the vector. These vaccines are designated ‘**t**ransmission **b**locking **v**accines’, or TBVs. Here, we summarize previous TBV work, discuss current research on arboviral TBVs and present several promising TBV candidates.

## Background

Infectious disease represents one the leading causes of mortality worldwide but it is especially problematic in tropical countries [1]. Among all infectious agents, viruses have been responsible for at least three major pandemics: smallpox, the “Spanish flu” (influenza virus) and the ongoing HIV/AIDS (human immunodeficiency virus) epidemic with more 30 million people affected globally [2, 3]. Smallpox infection was one of the major causes of death worldwide, with a death toll between 300 and 500 million people. In the late 1700s, Dr Edward Jenner pioneered an immunization practice, which eventually led to the eradication of this disease in 1980 [4, 5]. The principle of the smallpox vaccine and other viral vaccines is to educate the body with a non-pathogenic but closely related microorganism. Consequently, this establishes immunological memory, strengthens the immune system and prevents future attacks from the invading virus [6, 7].

Improving living conditions plays an important role in decreasing the global incidence of viral diseases but the implementation of vaccines has also had a significant impact [8, 9]. To date, several viral diseases have been controlled with the help of vaccines and educational campaigns. In the early 2000s, measles and rubella were eliminated from the United States and had lower incidence rates worldwide due to aggressive vaccination efforts [10, 11]. However, the re-emergence of mutant and drug resistant strains still poses a threat for the eradication of diseases like polio [12, 13], especially in areas with low vaccination coverage due to religious beliefs, lack of educational campaigns or general mistrust of health care workers providing the vaccines [13, 14]. Consequently, proper and timely education of the public is of pivotal importance in assuring and maintaining the efficacy of vaccination programs [15]. Vaccine efficacy and safety has improved considerably since Dr Jenner’s rudimentary smallpox vaccine through the use of several new technologies. These developments include the production of live/attenuated, inactivated, toxoids and subunit/conjugate vaccines aiming to induce long lasting immunological memory in order to respond quickly to the invading pathogen [7].

The majority of available vaccines protect against communicable viral diseases; however, for the vast majority of ‘arboviruses’, the name given to viruses transmitted by arthropods (arthropod-borne viruses), there is no vaccine alternative yet. The exceptions of this rule include the 17D yellow fever virus (YFV) vaccine and the newly approved dengue virus (DENV) vaccine, Dengvaxia [16, 17]. For decades, the main control method of arboviruses has relied on insecticide treatment of houses, reduction of breeding sites and limiting the vector-host contact (i.e., using a barrier protection like bed nets) [18–20]. New control strategies are needed due to increased insecticide resistance and the spreading of infectious arthropods to new areas [21, 22]. Unfortunately, insecticide resistance has spread to most countries endemic for vector-borne diseases [23]. Insect genetics and the continued/indiscriminate use of insecticides have largely contributed to the selection of resistant arthropods threatening the success of disease control programs [24, 25]. Fortunately, extensive research has shed some light onto the mechanism leading to resistance. For instance, insects can become insensitive to insecticide by metabolic resistance (metabolizing the insecticide faster than insecticide-sensitive strains), target modification (the insecticide-target molecule may change the structure/sequence) or behavioral modifications (indoor feeders changing to outdoor feeders) [26–28]. The presence of resistant mosquitoes in an area endemic for vector-borne diseases have a deleterious impact on public health control programs aimed to protect the population for exposure to infective arthropod bites [25]. Vector insecticide resistance also has a negative impact on economies as it pushes governments to use different and more expensive insecticides as well as requiring continuous re-treatment of areas [29, 30].

**Fig. 1:**
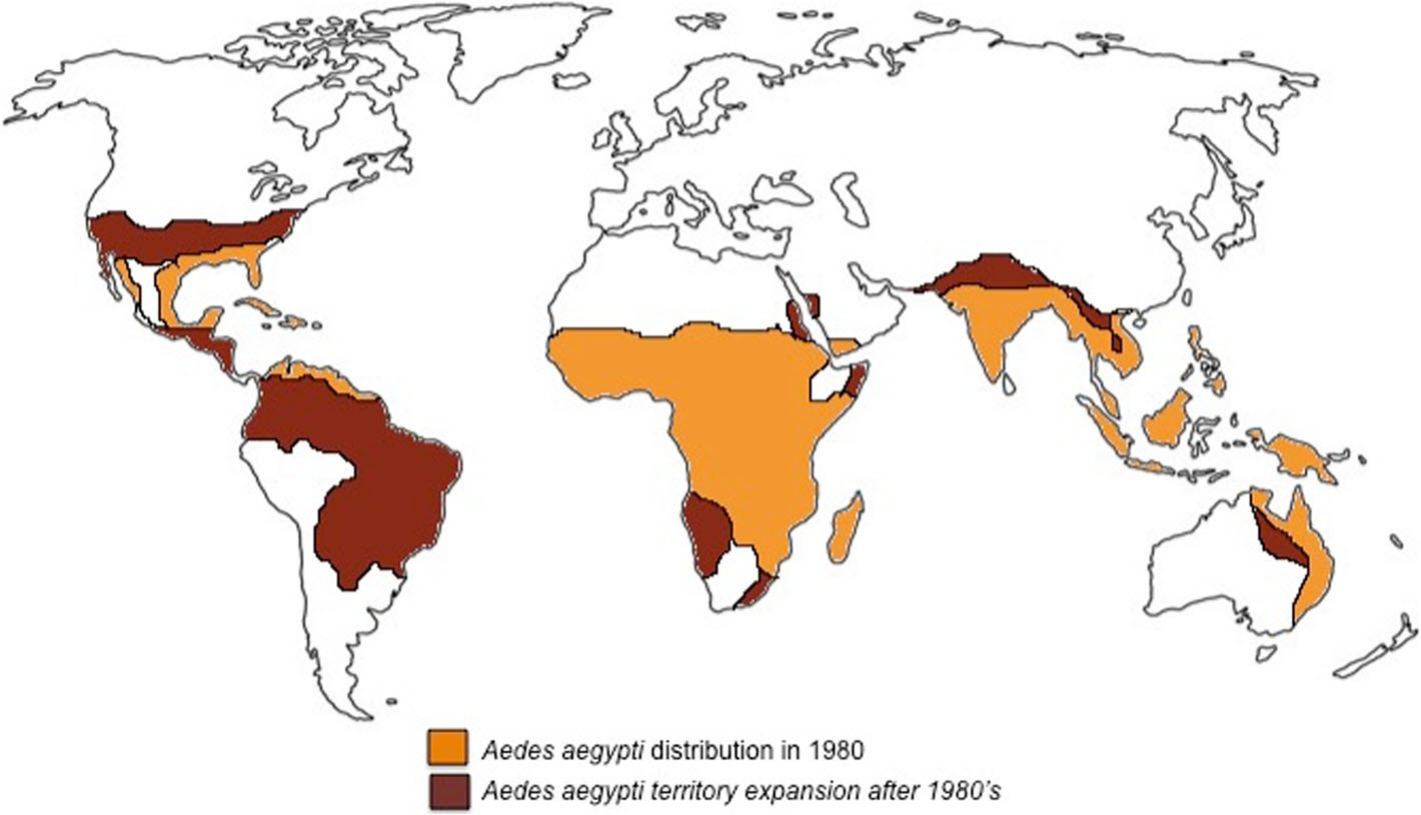
Schematic representation of the *Aedes aegypti* mosquito geographical expansion from 1980 to 2016

Climate variability has a direct impact on disease transmission since the life-cycle of the arthropod vector as well as the pathogen extrinsic incubation periods (EIPs), which depend on factors like temperature and humidity [31, 32], For instance, an increase in temperature has been associated with shorter extrinsic incubation periods and higher vector mortality [33–35]. Changes in vegetation and precipitation may induce an increase in mosquito densities in such a way that both factors are important predictors of mosquito abundance [36, 37] An example of the expansion of *Aedes aegypti* mosquitoes towards more temperate regions is depicted in Fig. 1 [38–40]. Tick life-cycle is also affected by climate fluctuations, helping several species to expand its territory to new higher altitudes due to warmer seasons [41]. Likewise, environmental and social factors also have a great impact on vector-borne diseases, associated with land use, water storage and seasonal work along with global travel all contribute to the rapid movement of human carriers and infected mosquitoes worldwide disseminating diseases [42–44].

Designing vaccines that prevent the pathogen from completing its life-cycle in the vector is one approach to halting transmission to humans [45]. Such vaccine alternatives have received the name of **t**ransmission **b**locking **v**accines (TBVs). Different from traditional vaccines, TBVs aim to prevent infection in the transmitting vector rather than in the human host. They are also known as “altruistic vaccines”, where the person receiving the vaccine may or may not be protected from infection but may prevent their neighbors from getting the disease [46, 47]. Interestingly enough, protecting one’s neighbor could actually, in the end, prevent one’s own new infection due to a phenomenon called “herd immunity” [48]. Since TBVs will have long-term effects they have to meet certain criteria to be widely accepted and implemented in the field. For example, besides having a high efficacy of infection prevention to the arthropod vector, care must be taken to avoid any cross-reactions or autoimmune disease in humans [49]. In addition, one of the most difficult challenges for TBVs is the maintenance of high antibody titers in vaccinated individuals due to the lack of natural immunological boosting since majority of the proposed antigens are not normally found in the human but in the mosquito or tick vector [46, 47]. One way to solve this problem is through the use of adjuvants. Thus, significant effort has been put towards the design and implementation of new and safe adjuvants compatible with the vaccine formulations against mosquito, tick, or pathogen proteins [50, 51]. Adjuvants like the nanoparticle-forming exoprotein from *Pseudomonas aeruginosa A* (EPA), the outer-membrane protein complex (OMPC) of *Neisseria meningitidis* serogroup B and the IMX313, a chicken complement C4b-binding protein oligomerization domain, have shown to significantly increase antibody responses against the conjugated peptides [52, 53]. A substantial review on other adjuvants and their mechanism of action can be found in Bergmann-Leitner et al. [54].

There is a growing concern regarding emerging and re-emerging arboviruses in many parts of the world. In the last decade, the American continent has suffered the introduction of several mosquito-transmitted viruses. In the late 1990s, West Nile virus (WNV) was introduced to New York City. Recently, Central and South America have been the focus of the latest chikungunya virus (CHIKV) and Zika virus (ZIKV) epidemics [55, 56]. Interestingly, several TBVs targeting the transmission of malaria are currently in clinical trials [57, 58], suggesting that TBVs are a feasible method for limiting the spread of insect-borne diseases such as those caused by arboviruses. TBVs are an attractive tool to decrease arbovirus transmission, especially in the absence of specific antiviral treatments to prevent severity in high-risk populations, such as the elderly and pregnant women. The aim of this paper is to summarize the current TBV candidates for arboviruses and the most promising molecules that could be used in future TBV vaccine developments.

## Potential TBV impact on arboviral transmission

There are approximately 3.5 billion people at risk of contracting diseases transmitted by arthropods. More specifically, one sixth of the total infectious diseases worldwide are spread by arthropod vectors [59]. There are even some cases where a single arthropod species is able to transmit more than one human pathogen in a given time. This is the case of *Aedes aegypti,* a prolific vector of many arboviruses including DENV, CHIKV, ZIKV and YFV [60–62]. Thus, a tool targeting transmission capacity in the vector rather than a single anti-microorganism human vaccine could be a practical control method for these diseases [60, 63].

**Fig. 2:**
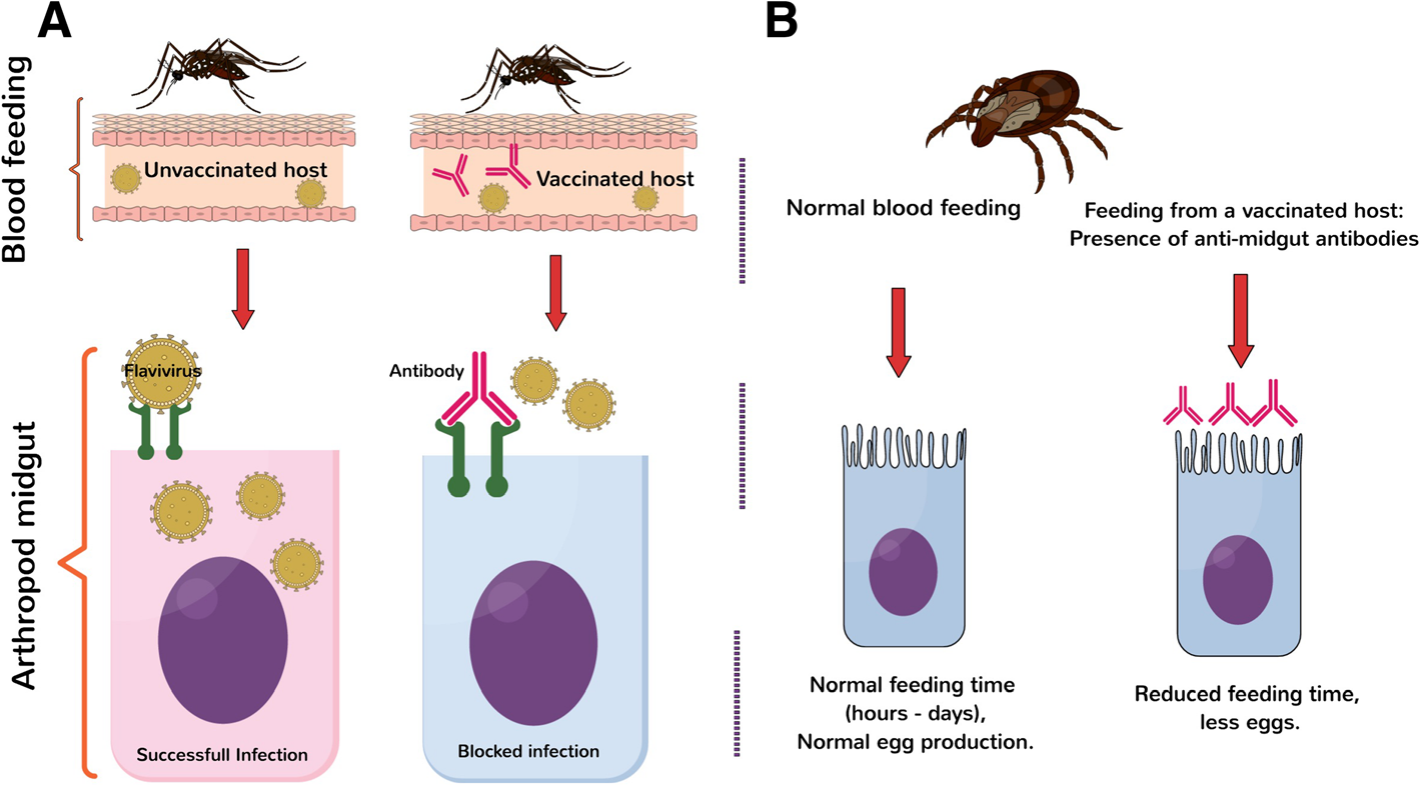
Representation of the principal mechanisms of TBVs. **a** In the case of TBVs based on the vector proteins that interact with the pathogen, the vector will ingest antibodies against the target protein during blood feeding inhibiting the pathogen entrance to cells. **b** In second type of TBV, antibodies are directed against proteins required for blood digestion or egg production. Thus, when the arthropod feeds on a vaccinated host, those antibodies may kill the vectors or decrease their capacity to laid eggs

Since the objective of the TBV vaccine is to halt new infection in transmission-competent insects, several approaches have been implemented to prevent infection in the vector (Fig. 2). To specify, most current TBV candidates focus on specific arthropod proteins while several others target pathogen antigens [64]. For instance, potential TBVs against DENV and WNV infections are designed using mosquito proteins required for viral infection of the mosquito vector [65, 66]. Some of these proteins have shown to be essential for virion attachment to the target cells or for successful completion of the viral life-cycle [67]. Conversely, other TBV candidates consist of molecules required by the vector to obtain or digest nutrients [68, 69]. Arthropod proteins carried by all members of the species may have an effect on vector feeding or physiological changes as well as impacting transmission capacity of all or several pathogens [70]. A summary of the most promising arthropod antigens for TBVs can be found in Table 1.

**Table 1:** Main arthropod protein candidates for TBVs to control arboviruses

Candidate molecule	Arthropod host	Function	Effect on disease	Available as vaccine
mosGCTL-1	*Aedes aegypti*	Involved in WNV attachment by interacting with PTP-1 [65]	Reduces WNV infection in mosquitoes	No
mosGCTL-3	*Aedes aegypti*	Modulates virus entrance by interacting with the DENV Envelope protein [67]	Reduce DENV infection in mosquitoes	No
CRVP-379	*Aedes aegypti*	Interacts with the putative DENV receptor prohibitin [63, 102]	Blocks DENV infection in midgut	No
CPB-1	*Aedes aegypti*	Midgut antigen. Interacts with the DENV Envelope protein [100, 101]	Reduces DENV infection in mosquito	No
64-TRP	*Rhipicepalus appendiculatus*	Salivary antigen. Secures ticks mouth parts during blood feeding [136, 137]	Reduces vector-host contact. Induces death of blood feed ticks	No
Bm-86	*Rhipicephalus microplus*	Midgut antigen [131, 134]	Reduces blood uptake and vector-host contact	Yes
PpChit-1	*Phlebotomus papatasi*	Aids maturation of peritrophic matrix [159]	Reduced sand fly life span and fecundity	No

## TBV to control arbovirosis transmitted by mosquitoes

### West Nile virus

WNV is a zoonotic arbovirus member of the *Flavivirus* genus and it is naturally maintained in an enzootic cycle between birds and mosquitoes [71]. Birds are the natural reservoir for WNV able to develop a transient high viremic titer allowing transmission of WNV to mosquitoes that preferentially feed on these birds. Migratory birds are of importance since they can carry the virus from one region to another [72]. Most bird infections with WNV results in non-fatal outcomes conferring a permanent immunity after infection [73]. However, several bird species, specially from the family Corvidae, do not usually survive WNV infection and are useful in tracking virus spread through dead-bird surveillance programs [74].

In addition, WNV virus infects horses and can be transmitted to humans in close contact with these animals [75]. Humans and horses are infected as incidental dead-end hosts with insufficient viremia to perpetuate the transmission cycle, but this virus is able to cause small outbreaks in naïve populations [76]. Before 1990, this disease occurred sporadically as a minor risk for humans. The first cases of severe WNV were reported between 1994 and 1996 during the Algeria and Romania outbreaks where neurological complications were reported in several cases [77, 78]. WNV has now spread globally and although majority of infections are asymptomatic, elder populations as well as immune-compromised individuals are at high risk of severe disease [79]. The incidence of WNV has also increased worldwide in the last decade and now is the major cause of human encephalitis in USA [80, 81]. One of the major epidemics in USA occurred in 2012 with more than 5000 human cases reported in 48 states that resulted in 286 deaths [82].

Several species are important vectors of WNV in USA, with *Culex pipiens* as one of the most important vectors, followed by *Culex quinquifasciatus* and *Culex tarsalis* [83]. However, at least 59 mosquito species have been identified as potential vectors and ten species have been confirmed as important vectors [84]. Consequently, continued surveillance as well as preventive methods are in need to control WNV infections in USA and worldwide.

Recent studies have demonstrated the role of C-type lectins in the establishment of *Flavivirus*, including WNV infection, in both mammals and the mosquito vector [85, 86]. For instance, Cheng et al. (2010) demonstrated that the knockdown of mosGCTL-1, or the feeding of mosquitoes with specific antibodies against the *Aedes aegypti* protein mosGCTL-1, significantly reduced WNV infection in both *Aedes* and *Culex* mosquitoes [65]. An upregulation of this protein is induced upon infection with WNV [65]. This study also showed that the *Ae. aegypti* C-type lectin (mosGCTL-1) interacts with WNV in a way that facilitates infection. MosGCTL-1 protein is recruited by mosPTP-1, an *Ae. aegypti* mosquito homolog of human CD45, which is a member of the of the protein tyrosine phosphatase (PTP) family. The interaction between mosGCTL-1 and mos-PTP-1 allows viral attachment to cells and enhances viral entry. Furthermore, both proteins are critical for WNV infection as demonstrated in experiments with *Culex quinquifasciatus*, the most competent vector for this virus [67]. Taking all this information together, mosGCTL-1 could be a potential target for a TBV against WNV.

### Dengue virus

Dengue is currently one of the most important viral diseases of the tropics causing high morbidity and mortality in pediatric populations [87, 88]. There are four distinct DENV serotypes, DENV 1–4, all transmitted by *Aedes* spp. mosquitoes. While genetically closely related, each serotype differs in antigenicity, thus cross-protection is limited [89–91] and infection with any serotype induces lifelong immunity to only that specific serotype [89, 92]. While the majority of DENV infections result in little or no disease, a small proportion progresses to severe forms: dengue with warning signs and severe dengue (WHO 2009) also known as dengue hemorrhagic fever (DHF) and dengue shock syndrome (DSS) [93]. The etiology of severe dengue is not completely understood; however, one of the most accepted theories suggests that dengue pathogenesis is related to antibody-enhanced infection (ADE) of DENV in Fc-receptor bearing target host cells [90]. Epidemiological studies suggest that antibodies to each of the four DENV are capable of enhancing further DENV infections. This phenomenon has been studied extensively in vitro and modeled in mice [94–97] and it is currently one of the challenges for the implementation of a protective vaccine against DENV infection.

An effective TBV for DENV should be able to block infection of all serotypes to prevent an increase in the severity of cases. Recent research has discovered several mosquito molecules with a critical role in DENV cell entry and replication [98, 99]. As seen in WNV, several C-type lectins have been associated with *Ae. aegypti* and DENV infection. For instance, as mosGCTL-1 mediates the attachment of WNV on cell membranes, the *Ae. aegypti*, mosGCTL-3 is involved in DENV-2 infection. A recent study showed that mosGCTL-3 interacts directly with DENV envelope protein (Ep) and its gene expression is significantly upregulated upon DENV2 infection. After immunization, antibodies directed against mosGCTL-3 were able to disrupt mosquito infection with DENV-2 [67]. This indicates that GCTL-3 may be a suitable candidate for the development of a TBV.

Several suitable avenues in the search for TBV targets in mosquitoes have been explored. One approach, protein interaction screening, revealed that the *Ae. aegypti* carboxypeptidase B1 (CPB1) is one of the predominant midgut proteins interacting with the DENV-2 Ep [100]. Previous studies suggested that CPB1 is upregulated after a blood meal and it is involved in the intra- and extracellular accumulation and secretion of infectious DENV-2 particles [101]. CPB1 was found throughout the cells but the Ep-CPB1 complex was only observed in the endoplasmic reticulum close to the nuclei. Tham et al. (2014) suggested that CPB1 is involved in regulating viral replication and the release of virion particles from midgut cells [100]. In the presence of CPB1, virus released from infected cells is predominantly immature, decreasing the chances for the virus to colonize salivary glands, and consequently reducing virus transmission to humans and other mammals [100].

Another protein candidate for a TBV against DENV infection is the putative cysteine rich venom protein 379 (CRVP379). CRVP379 expression is also upregulated by infection with DENV. Our group recently published that silencing CRVP379 significantly reduced DENV infection in *Ae. aegypti* mosquitoes. Additionally, we have found that CRVP379 is required for DENV infection and that it directly interacts with prohibitin, a putative receptor for DENV in *Aedes* spp. Blocking CRVP379 with specific antibodies recognizing the mosquito protein significantly reduced DENV infection in mosquitoes [66]. Additionally, CRVP379 has very low homology to any mammalian or human proteins. Since CRVP379 was found to be upregulated in WNV and YFV infections, we speculate that this protein is also required for infection of mosquitoes with these diseases; thus, antibodies against CRVP379 may also impact infection and subsequent transmission of other closely related flaviviruses [102]. If this hypothesis is correct, a TBV based on CRVP379 could have an enormous impact in places with concurrent transmission of these arboviruses, which is the case in most South and Central American countries. We are currently testing this hypothesis in our laboratory.

### Rift Valley fever virus

Rift Valley fever virus (RVFV) is mainly maintained in the environment by transmission between animals (wild or domestic) and several mosquito species including *Aedes*, *Culex* and *Anopheles* [103, 104] although occasional transmission by other hematophagous insects has been reported [105, 106]. Most human cases of RVFV are mild; however, a small proportion progresses to the severe forms of the disease manifesting as meningoencephalitis or hemorrhagic fever [107, 108].

Although no TBV vaccine has been evaluated to control RVF infections, significant advances have been made in the biology of the mosquito vectors trying to block transmission of other diseases. For instance, the most promising *Anopheles* protein with potential of impacting RVFV transmission to date is the midgut protein alanyl aminopeptidase N (AnAPN1) [109]. This protein is present on the *Anopheles gambiae* midgut apical surface and it is believed to function as a receptor for *Plasmodium* ookinetes. Antibodies against AnAPN1 are able to inhibit *P. falciparum* parasite load [110]. Further investigation is needed to evaluate the effect of this protein in the development of RVFV in *Anopheles* and its further transmission to the vertebrate host.

## TBVs with the potential for targeting multiple diseases transmitted by other arthropods

The blockage of arthropod proteins by the antibodies elicited against them through vaccines may have an impact not only on the lifespan of the arthropod vector but also on the transmission of most, if not all, microorganisms they carry [111, 112]. These are vector-derived molecules involved in food digestion or feeding capacity [64]. Following, we present a list of candidates developed with the purpose of decreasing vector-host contact or halting midgut infection and salivary gland invasion.

### Ticks

While mosquitoes represent the main vectors of human disease, ticks can also transmit several human pathogens and are the most important transmission vectors in veterinary medicine, serving as principal vectors of zoonotic diseases [113]. In blood feeding arthropods, midgut protein targets are preferred as this is the first organ in contact with blood factors and the newly invading pathogens [114, 115]. In mosquitoes, blood can remain in the midgut for at least 24 h before all factors start degrading due to the action of digestive enzymes. On the contrary, tick blood digestion may take a few days [116]. Several tick species can feed and regurgitate during the blood meal uptake process increasing the chances of pathogen transmission, especially because undigested blood in the tick midgut can be stored for long periods allowing enough time for the virus to interact with epithelia [117–120]. Different from other blood-sucker arthropods, tick feeding process is characterized by digestion of blood at the intracellular vesicles of gut cells [121]. Since this phenomenon is special characteristic of ticks, there are current directed towards the characterization of digestive enzymes and feeding regulation that could lead to new alternative for TBV design [122, 123].

Tick infestation represented a significant nuisance for the cattle industry. Therefore, a great deal of effort was put into the design of non-chemical insecticides and a few vaccines are commercially available. For instance, *Rhipicephalus* spp. (formerly *Boophilus* spp.) is a major cattle pest in tropical and subtropical countries [124, 125]. Infestations with *Rhipicephalus microplus* (cattle tick), severely impacts the cattle by inhibiting weight gain and decreasing milk production [125, 126]. Although there are reports that this species has been eradicated from most of USA, there are still cases of infestation leading to quarantine in bordering regions of Texas and California with Mexico [127]. Constant surveillance is implemented by the USDA Cattle Fever Tick Eradication Program to avoid the incursion of *R. microplus* into USA. The program consists of frequent patrolling/inspection of ranches in the Rio Grande area as well as acaricide prophylactic treatment and detention of smuggled livestock from Mexico [127, 128].

*Rhipicephalus microplus* is an important vector of *Babesia bovis* and *Anaplasma marginale* infections [129, 130]. In 1989, Willadsen et al. launched a new concept for the treatment of tick infestation. They described a tick gut antigen protein, Bm-86, and found that antibodies raised against this protein were able to reduce blood uptake and egg production of *Rhipicephalus* ticks [131]. In the following years, Gavac™ (Latin America) and TickGARD (Australia) vaccines based on the Bm-86 protein were commercially available. Antibodies against Bm-86 bind to the surface of the tick’s intestinal epithelial cells, which causes cell lysis. This leads to a reduction in the reproduction efficiency of *Rhipicephalus* ticks [132]. This vaccine also induces comparable antibody levels in different cattle breeds [133]. Interestingly, after the implementation of Gavac™ in Cuba, several studies showed a significant reduction in the incidence of tick-borne cattle diseases and subsequently, mortality [134, 135]. Currently, TickGARD and Gavac™ vaccine use is restricted to a small group of countries. However, effectiveness of these vaccines is restricted to *Rhipicephalus* ticks and efficacy may vary among tick strains [70, 135]. The development of new vaccines targeting other tick genera is needed.

Several anti-tick vaccine alternatives have been proposed. One of them is a vaccine based on a 15-kDa tick saliva protein, 64TRP. The 64TRP acts like a ‘cement’ and helps the tick to secure the mouthparts on the host skin during feeding [136]. This protein was described in the tick species *Rhipicephalus appendiculatus* and it induces a strong humoral response [68]. In vivo studies in rodents have shown that antibodies against 64-TRP not only impair tick feeding but also cross-react with midgut antigens, resulting in the death of engorged ticks. Furthermore, antibodies against 64-TRP have shown to be effective against both adult and immature stages of several tick species [136, 137]. Labuda et al. [137] demonstrated that 64-TRP induces an immune response suitable for a TBV that has protective functions comparable to TickGARD against TBEV. Other targets have also been proposed to control tick infestation and prevent disease transmission. For a more complete catalogue of anti-tick vaccine alternatives please review the study by Merino et al. [111].

Another tick of medical importance in USA is the lone star tick, *Amblyomma americanum,* a common parasitic arthropod of cattle and dogs among other species [138, 139]. This species is an important vector of Heartland and Bourbon virus, two emerging pathogens casing severe disease in humans in several regions within USA [140, 141]. One study was found in the literature addressing the possibility of a TBV vaccine to prevent transmission of diseases by the lone star tick. In this study, de la Fuente et al. [142] identified several potential TBV candidates by RNAi-based screening of the *A. americanum* cDNA library. The most promising candidates were the subolesin and the interphase cytoplasm foci protein 45, 2G7. Cattle vaccinated with these proteins showed a significant antibody production and reduction in tick infestation [142]. Due to the alarming increase of cases by Heartland and Bourbon viruses, more investigation is needed into potential vaccine targets that would block their transmission to and from the vector.

Previous studies have shown that certain vertebrate hosts have the ability to develop anti-tick immunity after several exposures to a particular tick species [143]. Based on this principle, the European group, the ANTIDotE consortium, was created to develop TBVs against *Ixodes ricinus* to fight the diseases transmitted by this tick species. In contrast to the anti-*Rhipicephalus* vaccines directed against the arthropod gut, ANTIDotE vaccines are based on salivary antigens that have showed an impact on vector feeding behavior and pathogen transmission [144].

In USA, *Ixodes scapularis* is known as an important vector of Powassan virus (POWV) the causing agent of Powassan encephalitis endemic in North America and eastern Russia [145]. Mortality rate of Powassan encephalitis is below 11 %. However, infection with POWV may lead to severe neurological sequelae [145–147]. Incidence of Powassan encephalitis has increased in the last fifteen years but it is still a ‘neglected’ tick-borne disease in North America [148]. Most research on this tick species is directed to stop other bacterial or parasitic diseases transmitted by this species and yet there is little research on transmission blocking vaccine being designed to stop POWV transmission [149]. The ANTIDotE approach may also lead to POWV candidates, especially because, POWV is transmitted imbibed in tick saliva and transmission occurs in the first hours of blood feeding [150]. Since tick-borne diseases are in expansion, extensive research is needed to characterize the factors involved in tick-borne virus transmission to prevent new infections, control spread and avoid emergence into new areas.

### Sandflies and phleboviruses

Several arboviruses are transmitted by sandflies in both Old and New World countries [151–154]. These viruses belong to the genera *Phlebovirus*, *Vesiculovirus* and *Orbivirus* [155, 156]. Most research on sand fly-based TBVs is aimed to control leishmaniasis. However, it is possible that the use of candidate proteins involved in blood meal digestion, fecundity or life span of sandflies may also have an impact on other diseases transmitted by these arthropods [157, 158].

One example is chitinase 1 (PpChit-1), the midgut protein of *Phlebotomus papatasi*. This protein is involved in the maturation of the peritrophic matrix (PM), which is of pivotal importance in blood digestion [157]. Previous studies show that antibodies against this protein also cross-react with other midgut proteins from different *Phlebotomus* species [159]. Most importantly, treatment with antibodies against PpChit-1 negatively impacts the ability of sandflies to lay eggs. This is potentially caused by PM permeability changes induced by the attaching antibodies. These changes affect nutrient absorption and egg development [159].

## Conclusions

In this review, we provide examples of new advances in vaccine technology aimed to block or eliminate insect-borne infectious diseases. Arboviruses are expanding not only geographically but they are also able to invade and be transmitted by a wide range of arthropods. At present, there are no specific treatments and very few vaccines that prevent most human arbovirus infections. Currently, prevention of arbovirus infection mainly relies on insecticide treatments, reduction of breeding sites and using barrier protection, such as bed nets or insecticide-impregnated clothing; however, these methods, although moderately effective, are not enough. Recently we have seen Heartland, CHIKV, and ZIKV virus outbreaks expand to the Americas at a rapid rate. In this regard, TBVs have the potential to decrease infection among certain populations while increasing herd immunity, which makes them an attractive tool to combat pathogen transmission and proliferation.

TBVs directed against specific arthropod molecules have the potential to impact most of the pathogens transmitted by a single vector. As with insecticides, there is always the possibility of resistance development against TBV vaccines, especially if genes with high selective pressure are targeted. To avoid possible selection, TBVs should be implemented along with other types of transmission control methods such as bed nets and insecticides to reduce vector population. In addition, combining pathogen and arthropod key target molecules in the same vaccine formulation may increase TBV efficacy and will confer protection against infection and transmission.

Access to vaccination and coverage strategies are very important factors in determining the effect of TBVs in countries endemic for specific diseases. Developing countries with economically challenged health care systems will greatly benefit from TBVs. Developing safe and affordable vaccine alternatives, along with community education, will improve the chances of reaching a broad distribution and effective vaccine coverage.

In summary, although significant advances have been made towards parasitic vector-borne diseases like malaria and leishmaniasis, further research is needed in TBV development for viral vector-borne diseases. TBVs represent a viable alternative to protect not only humans but also livestock.

## Abbreviations

64TRP: Tick cement protein, truncated constructs of 64P; AnAPN1: Alanyl aminopeptidase N; CHIKV: Chikungunya; CPB1: Carboxypeptidase B1; CRVP379: Putative cysteine rich venom protein 379; CTL: C-type lectins; DENV: Dengue virus; POWV: Powassan virus; PpChit-1: *Phlebotomus papatasi* chitinase 1; PTP: Protein tyrosine phosphatase; RVFV: Rift Valley fever virus; TBEV: Tick-borne encephalitis; TBV: Transmission blocking vaccine; WNV: West Nile virus; YFV: Yellow fever virus; ZIKV: Zika virus
